# BI-RADS-Based Classification of Mammographic Soft Tissue Opacities Using a Deep Convolutional Neural Network

**DOI:** 10.3390/diagnostics12071564

**Published:** 2022-06-28

**Authors:** Albin Sabani, Anna Landsmann, Patryk Hejduk, Cynthia Schmidt, Magda Marcon, Karol Borkowski, Cristina Rossi, Alexander Ciritsis, Andreas Boss

**Affiliations:** Institute of Diagnostic and Interventional Radiology, University Hospital of Zurich, University of Zurich, 8091 Zurich, Switzerland; albin.sabani@gmx.ch (A.S.); anna.landsmann@usz.ch (A.L.); patryk.hejduk@usz.ch (P.H.); cynthia.schmidt@hotmail.de (C.S.); magda.marcon@gmail.com (M.M.); karol.borkowski@usz.ch (K.B.); cristina.rossi@usz.ch (C.R.); alexander.ciritsis@usz.ch (A.C.)

**Keywords:** breast neoplasms, mammography, neural networks, computer, machine learning, artificial intelligence

## Abstract

The aim of this study was to investigate the potential of a machine learning algorithm to classify breast cancer solely by the presence of soft tissue opacities in mammograms, independent of other morphological features, using a deep convolutional neural network (dCNN). Soft tissue opacities were classified based on their radiological appearance using the ACR BI-RADS atlas. We included 1744 mammograms from 438 patients to create 7242 icons by manual labeling. The icons were sorted into three categories: “no opacities” (BI-RADS 1), “probably benign opacities” (BI-RADS 2/3) and “suspicious opacities” (BI-RADS 4/5). A dCNN was trained (70% of data), validated (20%) and finally tested (10%). A sliding window approach was applied to create colored probability maps for visual impression. Diagnostic performance of the dCNN was compared to human readout by experienced radiologists on a “real-world” dataset. The accuracies of the models on the test dataset ranged between 73.8% and 89.8%. Compared to human readout, our dCNN achieved a higher specificity (100%, 95% CI: 85.4–100%; reader 1: 86.2%, 95% CI: 67.4–95.5%; reader 2: 79.3%, 95% CI: 59.7–91.3%), and the sensitivity (84.0%, 95% CI: 63.9–95.5%) was lower than that of human readers (reader 1:88.0%, 95% CI: 67.4–95.4%; reader 2:88.0%, 95% CI: 67.7–96.8%). In conclusion, a dCNN can be used for the automatic detection as well as the standardized and observer-independent classification of soft tissue opacities in mammograms independent of the presence of microcalcifications. Human decision making in accordance with the BI-RADS classification can be mimicked by artificial intelligence.

## 1. Introduction

Breast cancer (BC) is the most commonly diagnosed cancer among women. With an incidence of 12.3%, it constitutes the leading cause of cancer death (15.5%) in the female population [1]. It appears that the incidence for BC in industrialized countries is higher than in developing countries, partly due to lifestyle factors. Moreover, lower screening rates and incomplete reporting lead to the discrepancy of incidences [2]. The main risk factors of BC include female gender, older age, genetic predisposition, especially the presence of BRCA-1 or BRCA-2 mutations, hormonal changes and a previous diagnosis of ductal carcinoma in situ (DCIS). Besides non-modifiable risk factors, mammographic density is an independent risk factor for BC and is known to be inductive for modifiable risk factors, such as hormonal changes due to menopausal transition, lifestyle, obesity or excessive alcohol consumption [3,4,5]. As demonstrated in numerous large-scale randomized trials, mammography screening is known to reduce relative BC mortality by 20–31% [6,7,8,9]. However, mammography has its limitations. On the one hand, it is examiner dependent. A second reading is an established quality control tool; therefore, screening programs are often time-consuming and cost intensive. In addition, sensitivity is strongly reduced in dense tissue. Whereas the sensitivity in almost entirely fatty breast is reported to be 98%, it decreases to 48% in women with very dense breasts [10]. Moreover, a false positive rate of about 20% in screening programs reduces their efficiency, potentially resulting in overtreatment with consequences for those affected [11].

With the introduction of the BI-RADS classification of the American College of Radiology (ACR), a large degree of standardization was introduced in the assessment of mammograms. Based on morphological features, the BI-RADS classification is divided into six categories indicating the relative probability of malignancy: negative (1), benign (2), probably benign (3), suspicious (4), highly suggestive of malignancy (5) and known biopsy-proven malignancy (6). Depending on the BI-RADS category, different patient management is recommended. For benign findings, no further treatment is required, whereas for probably benign findings, follow-up after 6 months is recommended. In case of suspicious findings, a biopsy is typically performed to obtain tissue samples for histological examination [12].

In this study, we focus on soft tissue opacities in mammography, which are one possible phenotype of BC. According to the BI-RADS catalogue, certain characteristics of a soft tissue lesion, such as irregular shape, fuzzy, microlobulated or spiculated margins, and high density are suspicious for malignancy [12] Microcalcifications are one additional feature in the assessment of BC, then often indicative for malignancy. However, soft tissue opacities or lesions can present without associated features, e.g., microcalcifications or architectural distortion, making it quite challenging to detect them.

In the past, computer-aided diagnosis (CAD) systems have been used to assist radiologists in decision making or even to replace them. Previous CAD systems rely on hand-crafted features based on prior expertise and expert instructions. Approaches based on the selection of hand-crafted features that characterize geometry and textures have been proposed for the classification of masses. Within the burgeoning field of artificial intelligence, deep learning has been introduced as a new paradigm to interpret certain features directly from an image. To train deep convolutional neural networks (dCNNs) for breast cancer diagnosis, data samples need to be labelled, which enables the detection of complex associations in the data [13].

Previous studies have shown the applicability of a dCNN on mammographic images. For example, Becker et al. reported that a neural network was capable of detecting BC on mammograms with an accuracy similar to radiologists [14]. Using the detection and classification of breast lesions by ultrasound as an example, Ciritsis et al. demonstrated that their implemented dCNN with a sliding window approach similar to ours reaches high accuracy, thereby mimicking human decision making [15]. Different studies successfully trained a dCNN to classify microcalcifications according to the BI-RADS classification system, providing the expertise of a radiology team [16]. Even if the accuracy of the dCNNs is reported to be comparable to human readers, the process of decision making often remains obscure for the user.

The purpose of this study was to provide a dCNN where the decision making in the presence of BC is based on one single feature and is therefore comprehensible for the user. Therefore, the aim of this study was to train a dCNN for the classification and detection of breast cancer solely by its appearance as a soft tissue opacity without the presence of associated features such as microcalcifications.

## 2. Materials and Methods

### 2.1. Database Search

A local database search from the Picture Archiving and Communication System (PACS) was performed (A.S.). Between 2010 and 2019, 32,579 mammographies were performed at our institution. Based on the ethics proposal, data from the years 2010 to 2016 could be included in the study, as no signed informed consent had to be obtained from these patients. For the data from 2017 to 2019, only those were included who had given written consent to use the data for research purposes. According to the standards of our institution, all mammograms had undergone double-reading by two radiologists with over five years of experience in mammography. Based on the radiologists’ report, distribution was as follows: BI-RADS 1: 3626 examinations; BI-RADS 2: 22,727 examinations; BI-RADS 3: 4882 examinations; BI-RADS 4: 850 examinations; BI-RADS 5: 330 examinations; BI-RADS 6: 164 examinations.

**Figure 1:**
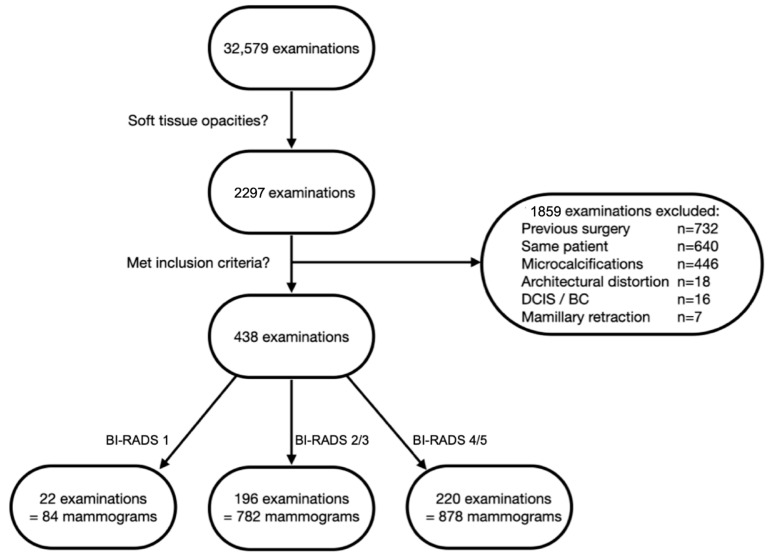
Flowchart depicting patient selection process. Only examinations depicting soft tissue opacities were included. Examinations of women with previous surgery, diagnosed DCIS or breast cancer (BC) on the side examined and examinations from the same patient were excluded. Soft tissue opacities with associated features, e.g., microcalcifications, architectural distortion or mamillary retraction, were excluded.

A full-text search for “soft tissue opacities” resulted in 2297 examinations from 1549 patients. Examinations from patients with previous surgeries (*n* = 732) and previous diagnosis of DCIS or breast cancer on the side examined (*n* = 16) were excluded. Further, any mammograms depicting soft tissue opacities with associated features, e.g., microcalcifications (*n* = 446), architectural distortion (*n* = 18) or mamillary retraction (*n* = 7) were excluded. From the remaining 1078 examinations, a single researcher (A.S.) retrieved 22 randomly chosen patients from the BI-RADS 1 cohort (84 mammograms), 196 from the BI-RADS 2 and 3 cohort (782 mammograms) and 220 from the BI-RADS 4 and 5 cohort (878 mammograms), resulting in a total of 1744 mammograms from 438 patients to train the dCNN. We divided the BI-RADS 4 and 5 cohort into two folders that were classified as “typically malignant”, which corresponded to spiculated lesions, and “not typically malignant”, which partly had criteria of benign lesions. The subdivision was based on a consideration of training the models with regard to this differentiation. However, this procedure was discarded in the further course, and the two folders were combined. The patient inclusion process is depicted in Figure 1.

Out of all the retrieved mammograms, 54 were left for comparisons with human readers, and all the rest were randomly split in 70%:20%:10% proportions for the training, validation and testing of the dCNN models, leaving 1183 for training, 338 for validation and 169 mammograms for model-testing purposes.

### 2.2. Data Preparation

**Figure 2:**
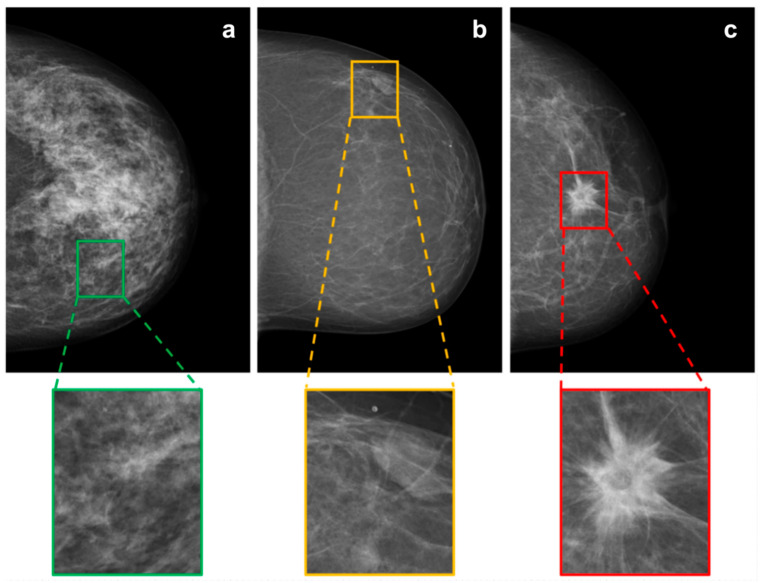
Mammographic illustrations of the three defined BI-RADS classes: (**a**) “healthy tissue”, (**b**) “probably benign soft tissue opacity” and (**c**) “suspicious soft tissue opacity”, with magnification of the ROI.

All mammographic images were initially resized to 3510 × 2800 pixels. A custom-made OCTAVE script (release 5.2.0) was used for the labeling task. According to three classes, for each mammogram, different rectangular regions of interest (ROIs) were manually labeled, cropped, and saved as new images (351 × 380 pixels). The classes were defined as 1: “healthy tissue” (BI-RADS 1), 2: “probably benign soft tissue opacities” (BI-RADS 2/3) and 3: “suspicious soft tissue opacities” (BI-RADS 4/5). For labeling healthy tissue, in Figure 2, mammographic illustrations of these three classes with magnification of the ROIs are shown. The human labeling and cropping of the original set of 1690 mammograms resulted in 7242 crops from images (see Table 1).

### 2.3. Training of dCNN Models

**Figure 3:**
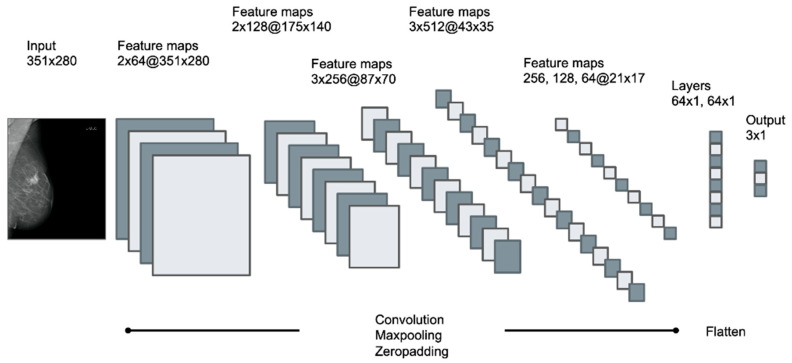
Schematic pattern of the applied multilayered deep convolutional neural network (dCNN), containing 13 convolutional layers with 4 max pooling layers followed by 2 dense layers. Input size of the image was 351 × 280 for dCNN models 1–3 and 175 × 140 for dCNN model 4. Feature maps are described as the number of layers x number of kernels at(@) the resolution of the images.

**Figure 4:**
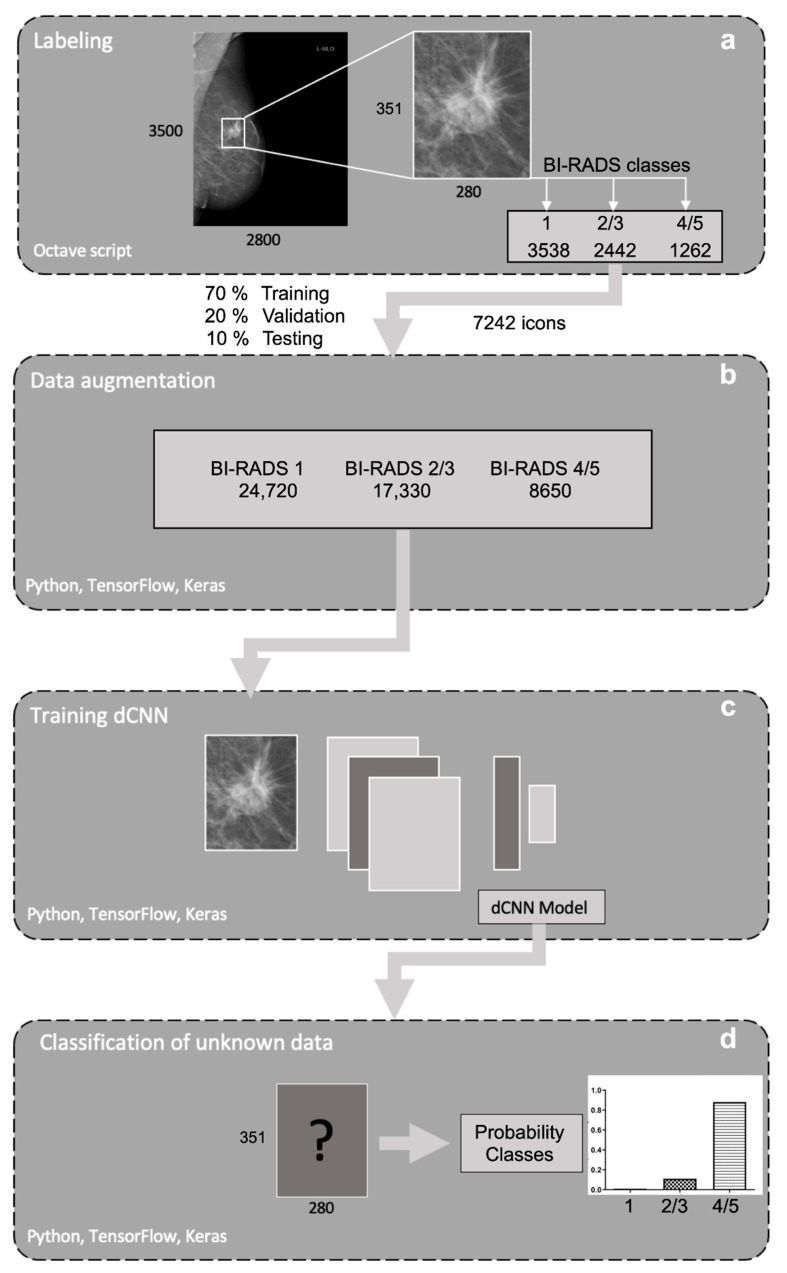
Schematic pipeline of the workflow used. (**a**) First mammograms were resized. For each image, different rectangular regions of interest (ROIs) were generated and labeled to three defined BI-RADS classes: “healthy tissue” (1), “probably benign opacities” (2/3) and “suspicious opacities” (4/5). (**b**) Data was randomly augmented with a built-in ImageDataGenerator class of Keras. (**c**) After image preprocessing and data augmentation, four different dCNN models were trained, validated and tested, (**d**) classifying the generated images based on the probability for the different BI-RADS classes.

Training was performed on a consumer-grade computer (Intel i7-7700, 16 GB, NVIDIA 1080 GTX 8 GB graphic card) running the operating system Ubuntu Linux 16.04. Models were created in the Python programming language (Python Software Foundation, version 3.8.24), using Keras 2.0.4 (Massachusetts Institute of Technology, Cambridge, MA, USA) for model compilation and training. Models were built with a sequential class of Keras, containing 13 convolutional layers with four max pooling layers for downsampling, followed by two dense layers with the ReLU activation function. To reduce overfitting, 50% dropout and l1/l2 regularization was applied, class weights were added to counteract class imbalance and a softmax activation function was used for the final weights. Models were trained with categorical cross-entropy loss function. To classify the three types of opacities described above according to the BI-RADS standard, four different dCNN models were generated, differing in the optimizer (SGD, Adam), batch size (16, 32), learning rate (1 × 10^−5^, 5 × 10^−5^) and input size of the image (351 × 280, 175 × 140). A graphical representation of the applied multilayered dCNN is shown in Figure 3. Training images fed to the network have been randomly augmented in 10-fold manner using the built-in ImageDataGenerator class of Keras with random shear, shift, zooming, rotating and brightness changes with the following settings: ‘zooming’: 0.05, ‘rotation’: 5.0, ‘horizontal_shift’: 0.05, ‘vertical_shift’: 0.05, ‘brightness’: 0.1′. Figure 4 depicts the schematic workflow from labeling to classification.

### 2.4. Human Readout on a “Real-World” Dataset

To evaluate unbiased performance, a “real-world” test dataset consisting of 54 mammograms not used during the training process was created. The images were classified based on the highest probability assigned to the different categories “probably benign soft tissue opacities” and “suspicious soft tissue opacities”. Diagnostic performance was then compared to human reading by two highly experienced radiologists in breast imaging (reader 1: 3 years of experience (C.S.); reader 2: 7 years of experience (M.M.)). For the evaluation with the dCNN, soft-tissue lesions were present in the center of all images (351 × 280 pixels), with 29 “probably benign lesions” (BI-RADS 2 + 3) and 25 “suspicious lesions” (BI-RADS 4 + 5). The radiologists received the entire mammography in one dimension for their reading. The performance of the dCNN model, as well as that of the two radiologists in terms of classification into the two categories “probably benign soft tissue opacities” and “suspicious soft tissue opacities”, was assessed using receiver-operating characteristics (ROC) in comparison to the clinical radiological reports, which served as the ground-truth.

### 2.5. Computation of Probability Maps

Representative mammographies were analyzed using a sliding window approach implemented with the computer vision OpenCV library (Intel Corporation, Santa Clara, CA, USA) Berkeley Software Distribution License). At each position of the sliding window with an increment of 10 in the x and y positions in the nested loops, a 351 × 280 array was cropped and classified with the dCNN model. The probabilities determined by the dCNN classification and the center coordinates were noted for each position of the sliding window. For visualization of the classification results, probabilities were converted into an RGB image according to the three classes (“healthy tissue”: blue, “probably benign lesions”: purple, “suspicious lesions”: red).

### 2.6. Statistical Analysis

The statistical evaluation was performed using IBM SPSS Statistics software (version 27.0, IBM Corp. Armonk, NY, USA). Inter-rater agreement between the dCNN, both the readers’ and the radiologists’ report (ground-truth) was calculated using Fleiss’ kappa. The strength of agreement beyond chance obtained can be interpreted as follows: poor, <0; slight, 0–0.2; fair, 0.21–0.4; moderate, 0.41–0.6; substantial, 0.61–0.8; almost perfect 0.81–1 [16]. For inter-reader reliability, an intraclass correlation coefficient (ICC) greater than 0.8 was considered almost perfect. The level of significance was set to a *p*-value < 0.05.

## 3. Results

### 3.1. Data Preparation and Training of dCNN Models

**Figure 5:**
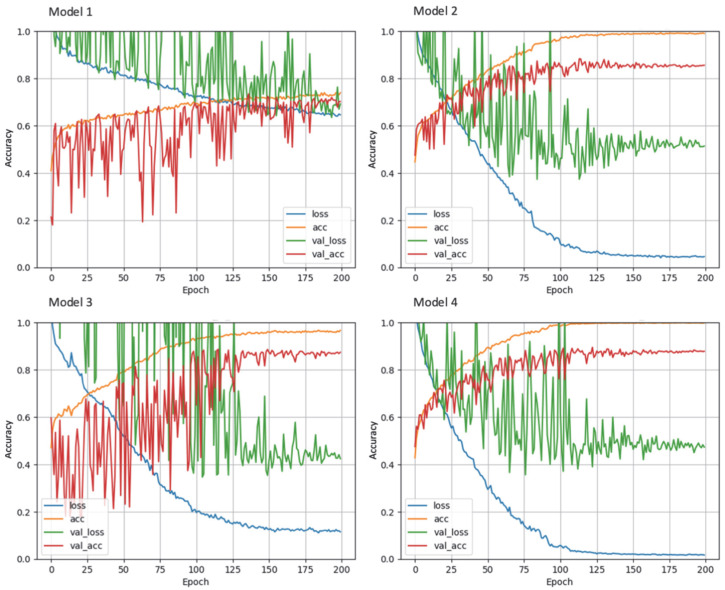
Learning curves of the different dCNN models (1–4) on the training and validation dataset. Accuracy is shown on the y-axis versus number of epochs on the x-axis. Model 1: SGD optimizer; model 2: ADAM optimizer; model 3: increased data augmentation; model 4: halved input size. The best performance was achieved by model 3.

Figure 5 shows the progression of the training and validation accuracies as well as the loss function for the different dCNN models. In model 1, a stochastic gradient descent (SGD) optimizer was used, and only moderate augmentation (zooming, rotating, horizontal and vertical shifting, brightness) of the images was performed, which resulted in the lowest accuracy of only 73.8% (95% CI: 70.4–76.9%). In model 2, the SGD optimizer was replaced by Adaptive Moment Estimation (ADAM), which resulted in an accuracy of 88.4% (95% CI: 85.8–90.6%), an improvement of 14.6% compared to the SGD optimizer. In model 3, data augmentation was increased, which resulted in the highest accuracy of all models of 89.8% (95% CI: 87.3–91.9). The fourth model, in which the matrix size of the input images was reduced by half, led to an accuracy of 88.4% (95% CI: 85.8–90.6%). For all models, the accuracy of the validation dataset was initially higher than that of the training dataset, which may be explained by the small batch size used for training, whereas the validation dataset is evaluated completely after each epoch. Confusion matrices for the test data set are shown in Table 2. For model 1, a systematic deviation of the dCNN prediction to lower classes can be seen, whereas in the other confusion matrices, a mostly symmetric behavior can be observed.

### 3.2. Validation on “Real-World” Data

**Figure 6:**
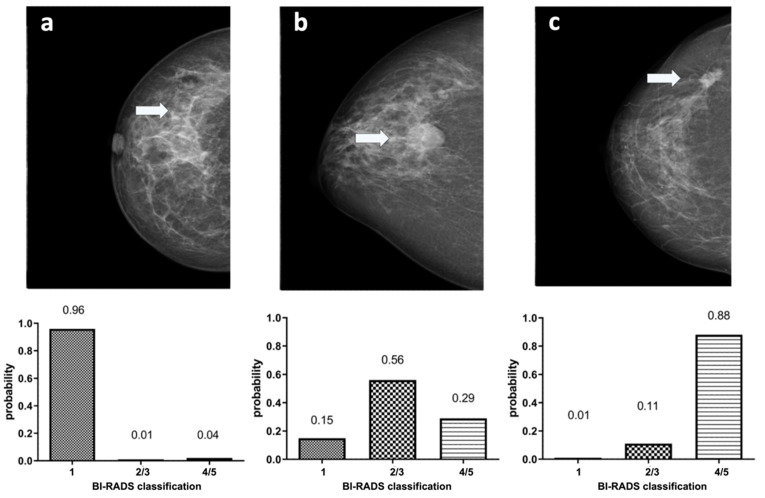
Example images showing (marked with an arrow) normal breast parenchyma (**a**), a probably benign soft tissue opacity (**b**) and a suspicious soft tissue opacity (**c**) with corresponding classification probabilities of dCNN model 3 to the three defined BI-RADS classes (1) “healthy tissue” (2/3) “probably benign soft tissue opacity” and (4/5) “suspicious soft tissue opacity”.

**Figure 7:**
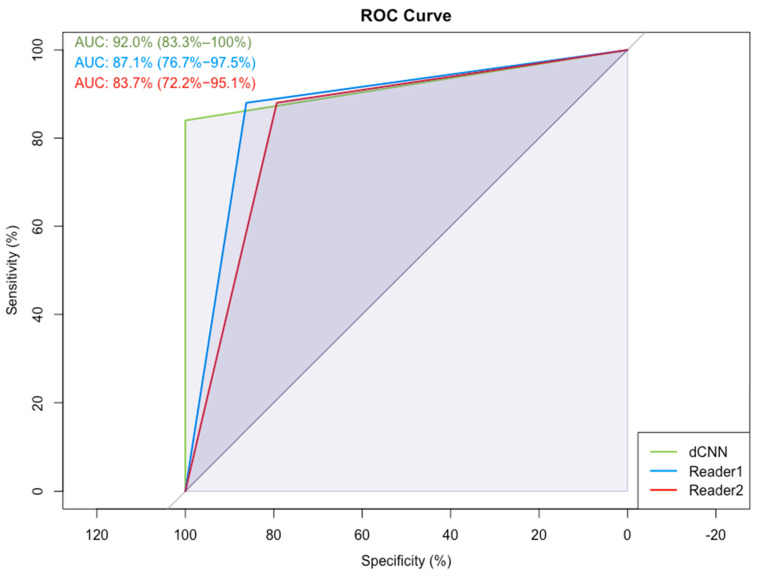
Receiver-operating characteristics (ROC) and the corresponding area under the curve (AUC) for dCNN model 3 and the two readers for the classification “probably benign soft tissue opacity” and “suspicious soft tissue opacity” compared to the radiologist’s report, which served as the ground-truth.

In Figure 6, example images for each class and the assigned probabilities are depicted. The confusion matrices of the “real-world” test dataset for dCNN model 3 and both human readers are presented in Table 3. In relation to the radiological reports, which served as a ground-truth, the dCNN reached an overall accuracy of 92.6% (95% CI:82.1–97.9). Four suspicious opacities were misclassified as probably benign, resulting in a sensitivity of 84.0% (95% CI: 63.0–95.5%), which was comparable to that of the radiologists (reader 1: 88.0%, 95% CI: 67.4–95.4%; reader 2: 88.0%, 95% CI: 67.7–96.8%). However, the specificity for the dCNN (100%, 95% CI: 85.4–100%) was superior compared to that of the radiologists (reader 1: 86.2%, 95% CI: 67.4–95.5%; reader 2: 79.3%, 95% CI: 59.7–91.3%. The inter-rater reliability between the three raters was substantial, with an ICC of 0.77 (95% CI 0.65–0.87, *p*-value < 0.001). Kappa values between both readers and the dCNN were moderate but almost perfect between the dCNN and the ground-truth (Table 4) [16]. The diagnostic performance of the dCNN was excellent, with an area under the receiver operating characteristics (ROC) curve of 92% (95% CI: 83.3–100%), which was superior to both readers (reader 1: 87.1, 95% CI 76.6–97.5%; reader 2: 83.7%, 95% CI 72.2–95.1%), as depicted in (Figure 7).

### 3.3. Probability Maps

**Figure 8:**
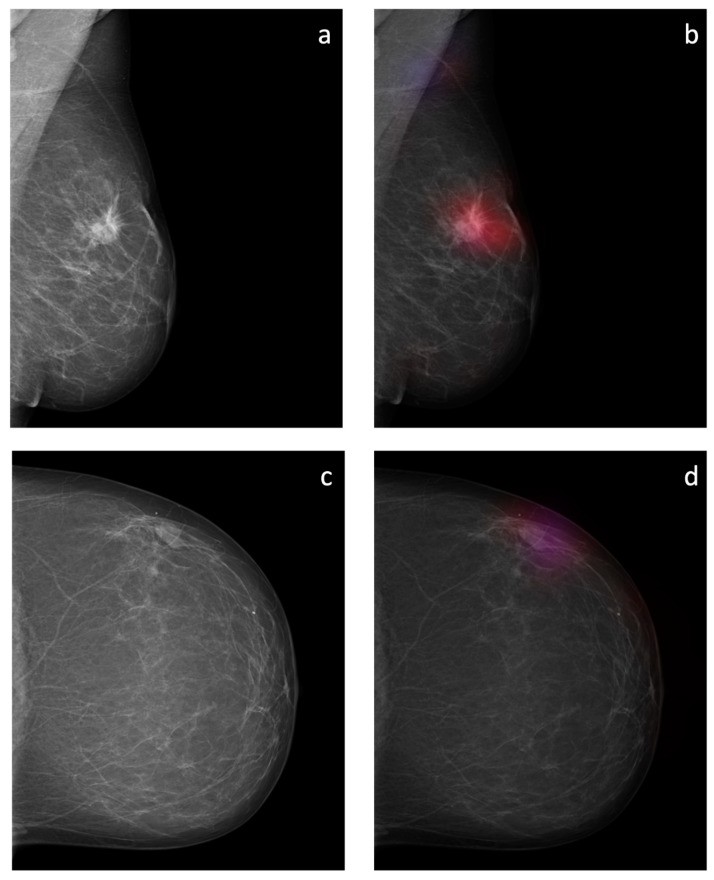
Overlay images of the calculated probability maps (**b**,**d**) created by the sliding window approach and the corresponding mammographic images (**a**,**c**) of two patients from the test dataset. In (**a**,**b**), a suspicious soft tissue opacity is highlighted in red. An area of denser tissue in the left axilla is correctly highlighted as a healthy tissue in blue. In (**c**,**d**), a probably benign soft tissue opacity is highlighted in purple.

The sliding window approach was able to correctly detect the areal distribution of the suspicious soft tissue opacity. Excellent image quality could be obtained. Examples of probability maps are shown in (Figure 8).

## 4. Discussion

In this study, we were able to show that artificial intelligence in the form of deep convolutional neural networks (dCNN) can be trained to distinguish between benign and malignant soft tissue opacities in mammograms on a BI-RADS based approach. Depending on the BI-RADS classification, a decision is made as to whether no further action is necessary (BI-RADS 1), a follow-up examination in 6 months is recommended (BI-RADS 2 and 3) or if a biopsy is indicated (BI-RADS 4 and 5). The proposed dCNN model was able to distinguish probably benign and suspicious findings solely based on the specific features of the soft tissue opacities without associated microcalcifications, asymmetries or architectural distortions. As the sensitivity of our dCNN was comparable to human readers, we were able to demonstrate that human decision making can be mimicked by the algorithm in regard of the assessment of soft tissue opacities, which are often a cause of uncertainty for inexperienced radiologists. Moreover, the specificity and accuracy of our dCNN was superior to that of the human readers, showing that artificial intelligence can be used as a second reading for mammographic images, providing a time-saving approach in screening programs.

There are several studies with various approaches regarding the role of machine learning and its capabilities to detect suspicious masses in mammograms. In a study from 2016, Lévy and Jain used deep learning (DL) to discriminate between benign and malignant regions in mammograms. They additionally put the masses in the context of the parenchyma surrounding the mass. With their approach, they were able to reach an accuracy of 92.4%, which was comparable to our study [12]. Another study by Shen et al. took the approach of assessing the complete image of the mammogram using DL, which was able to achieve an accuracy of 96% [17]. However, their aim was only to detect the lesion; the classification of the lesion was out of the scope of their study.

Whereas different studies often used DL to assess the image with all its features, we focused on detecting and classifying local soft tissue opacities without “typical” associated features, such as microcalcifications, architectural distortions, cutis thickening, enlarged lymph nodes or others. Not only are associated features often indicative for malignancy, but they also lead to attention on a suspicious area in the mammogram. Without the presence of microcalcifications or other features, detecting and assessing suspicious tissue opacities seems more challenging, particularly in the presence of surrounding glandular tissue. Since mammograms are superimposed images, the effect of tissue overlay impedes the visibility of tissue opacities, especially in women with denser breast tissue, reducing the sensitivity of screening programs [10]. The lack of additional features such as microcalcifications, architectural disturbances and cutis thickening may by the origin of the lower sensitivity of 87.9% observed in our study.

Deep convolutional neural networks as used in our study are currently the most powerful deep learning algorithm [18]. However, the training of a dCNN requires many data and a lot of computing power. Therefore, a specific adaptation of the neural network was necessary to achieve sufficient accuracy. The original optimizing algorithm “SGT” used in dCNN model 1 reached the lowest accuracy compared to model 2–4 using the more powerful optimizer “ADAM”.

Despite the presence of certain features, the assessment of mammographic images, particularly soft tissue opacities, strongly depends on the radiologists’ experience. In our study, the sensitivity of the dCNN was comparable to the human readers (87.9% vs. reader 1: 84.6% vs. reader 2: 78.6%). However, the specificity was perfect (100%), leading to a higher overall accuracy (92% vs. reader 1: 87.1% vs. reader 2: 83.7%). Artificial intelligence, therefore, may serve as a second reading tool to improve image assessment. Particularly in screening programs, where second reading is a standard procedure, AI algorithms could be a cost-effective alternative. However, in radiological imaging, the process of decision making by AI algorithms is often considered a black box, whereby the user knows the input and output but is not aware of the image features underlying the classification decision. This lack of information can be a problem for clinical applicability. Therefore, AI algorithms need to be trained to classify the different relevant features of breast cancer such as soft tissue opacities and microcalcifications (explainable AI). With the proposed technology, we will provide another element to improve the applicability of AI in breast imaging.

Our study has several limitations: First, compared to other studies applying AI on BC detection, only few data were used. Because our intention was to train the dCNN on the detection of soft tissue opacities, we omitted many mammographic images showing additional features of BC such as microcalcifications or architectural distortions. However, it was out of the scope of this study to train a dCNN with higher accuracy than previous studies. Instead, we wanted to provide the proof-of-principle that a dCNN can be trained with high accuracy for the detection of breast cancer solely by the feature of soft tissue opacities. Second, we cannot exclude that different machine learning algorithms other than dCNNs might reach a higher accuracy on the available amount of data. However, dCNNs are currently regarded as the most powerful machine learning algorithm [18]. Third, only four different dCNN configurations have been tested. From our initial optimizations of the dCNN, however, we know that optimizer, spatial resolution and the degree of data augmentation are among the most influential parameters. Fourth, we did not systematically evaluate how dense breast tissue influences the detectability of breast lesions in the mammography using our dCNN model, which is an interesting question that should be addressed in a different study. Fifth, we also did not evaluate how the presence of additional features such as microcalcifications might influence the performance of our dCNN model. However, as the aim of this study was to provide the proof-of-principle that a dCNN can be trained to detect breast cancer as soft tissue opacities without additional features, the testing of supplementary features was out of our scope.

In conclusion, we were able to show that a dCNN can be successfully trained to accurately classify soft tissue opacities on mammograms according to the BI-RADS classification system to obtain an observer-independent classification with the ability to provide a standardized recommendation for the follow-up procedure. In addition, we were able to highlight benign and suspicious soft tissue opacities in the mammograms using a sliding window approach. The proposed technique might be used as a standardized quality control tool, providing the expertise of a team of radiologists.

## Figures and Tables

**Table 1:** Number crops of mammograms used for training, validation and testing of the different dCNN models.

	Category		
	BI-RADS 1 (Healthy Tissue)	BI-RADS 2/3 (Probably Benign Opacities)	BI-RADS 4/5 (Suspicious Opacities)
**Training**	2472	1733	865
**Validation**	695	489	264
**Test**	371	220	133

**Table 2:** Confusion matrices of the different dCNN models (1–4) on the test dataset, with 1: “healthy tissue”, 2/3: “probably benign opacities”, 3/4: “suspicious opacities”. The correctly assigned classes are highlighted in bold.

**Model 1**	**Predicted**			
real		**1**	**2/3**	**4/5**
	1	**307**	36	3
	2/3	45	**191**	5
	4/5	27	74	**36**
**Model 2**	**Predicted**			
real		**1**	**2/3**	**4/5**
	1	**331**	11	4
	2/3	17	**215**	9
	4/5	23	20	**94**
**Model 3**	**Predicted**			
real		**1**	**2/3**	**4/5**
	1	**316**	14	16
	2/3	9	**222**	10
	4/5	13	12	**112**
**Model 4**	**Predicted**			
real		**1**	**2/3**	**4/5**
	1	**325**	13	8
	2/3	18	**208**	11
	4/5	18	12	**197**

**Table 3:** Confusion matrices for reader 1, reader 2 and dCNN model 3 for classification to BI-RADS 2/3, “probably benign soft tissue opacities”, and BI-RADS 4/5,“suspicious soft tissue opacities” on the “real world” dataset. Correctly assigned classes are highlighted in bold.

**Reader 1**	**Predicted**		
radiological report (ground-truth)		**2/3**	**4/5**
	2/3	**25**	4
	4/5	3	**22**
**Reader 2**	**Predicted**		
radiological report (ground-truth)		**2/3**	**4/5**
	2/3	**23**	6
	4/5	3	**22**
**dCNN**	**Predicted**		
radiological report (ground-truth)		**2/3**	**4/5**
	2/3	**29**	0
	4/5	4	**21**

**Table 4:** Inter-reader reliability between dCNN model 3, both readers and the radiologists report (ground-truth). Kappa values of 0.81–1.0 were considered almost perfect, 0.61–0.80 as substantial and 0.41–0.60 as moderate agreement.

	Ground-Truth	dCNN Model 3	Reader 1	Reader 2
Ground-truth	1	0.85 (CI: 0.71–0.99)	0.74 (CI: 0.56–0.92)	0.67 (CI: 0.47–0.87)
dCNN model 3		1	0.59 (CI: 0.38–0.80)	0.52 (CI: 0.30–0.74)
Reader 1			1	0.48 (CI: 0.25–0.72)
Reader 2				1

## Data Availability

The data presented in this study are available on request from the corresponding author. The data are not publicly available due to privacy restrictions.

## References

[1] Sung H., Ferlay J., Siegel R.L., Laversanne M., Soerjomataram I., Jemal A., Bray F. (2021). Global Cancer Statistics 2020: GLOBOCAN Estimates of Incidence and Mortality Worldwide for 36 Cancers in 185 Countries. CA Cancer J. Clin..

[2] Francies F.Z., Hull R., Khanyile R., Dlamini Z. (2020). Breast cancer in low-middle income countries: Abnormality in splicing and lack of targeted treatment options. Am. J. Cancer Res..

[3] Sun Y.S., Zhao Z., Yang Z.N., Xu F., Lu H.J., Zhu Z.Y., Shi W., Jiang J., Yao P.P., Zhu H.P. (2017). Risk Factors and Preventions of Breast Cancer. Int. J. Biol. Sci..

[4] Huang W.Y., Newman B., Millikan R.C., Schell M.J., Hulka B.S., Moorman P.G. (2000). Hormone-related factors and risk of breast cancer in relation to estrogen receptor and progesterone receptor status. Am. J. Epidemiol..

[5] Kamińska M., Ciszewski T., Łopacka-Szatan K., Miotła P., Starosławska E. (2015). Breast cancer risk factors. Przegląd Menopauzalny.

[6] Blanks R.G., Moss S.M., McGahan C.E., Quinn M.J., Babb P.J. (2000). Effect of NHS breast screening programme on mortality from breast cancer in England and Wales, 1990–1998: Comparison of observed with predicted mortality. BMJ.

[7] Otto S.J., Fracheboud J., Looman C.W., Broeders M.J., Boer R., Hendriks J.H., Verbeek A.L., de Koning H.J., Screening N.E.T.f.B.C. (2003). Initiation of population-based mammography screening in Dutch municipalities and effect on breast-cancer mortality: A systematic review. Lancet.

[8] Tabár L., Fagerberg C.J., Gad A., Baldetorp L., Holmberg L.H., Gröntoft O., Ljungquist U., Lundström B., Månson J.C., Eklund G. (1985). Reduction in mortality from breast cancer after mass screening with mammography. Randomised trial from the Breast Cancer Screening Working Group of the Swedish National Board of Health and Welfare. Lancet.

[9] Olsen A.H., Njor S.H., Vejborg I., Schwartz W., Dalgaard P., Jensen M.B., Tange U.B., Blichert-Toft M., Rank F., Mouridsen H. (2005). Breast cancer mortality in Copenhagen after introduction of mammography screening: Cohort study. BMJ.

[10] Kolb T.M., Lichy J., Newhouse J.H. (2002). Comparison of the performance of screening mammography, physical examination, and breast US and evaluation of factors that influence them: An analysis of 27,825 patient evaluations. Radiology.

[11] Hofvind S., Ponti A., Patnick J., Ascunce N., Njor S., Broeders M., Giordano L., Frigerio A., Törnberg S., Van Hal G. (2012). False-positive results in mammographic screening for breast cancer in Europe: A literature review and survey of service screening programmes. J. Med. Screen..

[12] D’Orsi C., Sickles E., Mendelson E., Morris E. (2013). American College of Radiology (ACR) Breast Imaging Reporting and Data System Atlas (BI-RADS atlas).

[13] Arevalo J., González F.A., Ramos-Pollán R., Oliveira J.L., Guevara Lopez M.A. (2016). Representation learning for mammography mass lesion classification with convolutional neural networks. Comput. Methods Programs Biomed..

[14] Becker A.S., Marcon M., Ghafoor S., Wurnig M.C., Frauenfelder T., Boss A. (2017). Deep Learning in Mammography: Diagnostic Accuracy of a Multipurpose Image Analysis Software in the Detection of Breast Cancer. Investig. Radiol..

[15] Ciritsis A., Rossi C., Eberhard M., Marcon M., Becker A.S., Boss A. (2019). Automatic classification of ultrasound breast lesions using a deep convolutional neural network mimicking human decision-making. Eur. Radiol..

[16] Schonenberger C., Hejduk P., Ciritsis A., Marcon M., Rossi C., Boss A. (2021). Classification of Mammographic Breast Microcalcifications Using a Deep Convolutional Neural Network: A BI-RADS-Based Approach. Investig. Radiol..

[17] Shen L., Margolies L.R., Rothstein J.H., Fluder E., McBride R., Sieh W. (2019). Deep Learning to Improve Breast Cancer Detection on Screening Mammography. Sci. Rep..

[18] Schmidhuber J. (2015). Deep learning in neural networks: An overview. Neural Netw..

